# Behavioral and Neural Signatures of Visual Imagery Vividness Extremes: Aphantasia versus Hyperphantasia

**DOI:** 10.1093/texcom/tgab035

**Published:** 2021-05-05

**Authors:** Fraser Milton, Jon Fulford, Carla Dance, James Gaddum, Brittany Heuerman-Williamson, Kealan Jones, Kathryn F Knight, Matthew MacKisack, Crawford Winlove, Adam Zeman

**Affiliations:** 1 Discipline of Psychology, University of Exeter, Exeter EX4 4QG, UK; 2 Cognitive Neurology Research Group, University of Exeter Medical School, College House, Exeter EX1 2LU, UK

**Keywords:** aphantasia, autobiographical, hyperphantasia, imagery, neuroimaging

## Abstract

Although Galton recognized in the 1880s that some individuals lack visual imagery, this phenomenon was mostly neglected over the following century. We recently coined the terms “aphantasia” and “hyperphantasia” to describe visual imagery vividness extremes, unlocking a sustained surge of public interest. Aphantasia is associated with subjective impairment of face recognition and autobiographical memory. Here we report the first systematic, wide-ranging neuropsychological and brain imaging study of people with aphantasia (*n* = 24), hyperphantasia (*n* = 25), and midrange imagery vividness (*n* = 20). Despite equivalent performance on standard memory tests, marked group differences were measured in autobiographical memory and imagination, participants with hyperphantasia outperforming controls who outperformed participants with aphantasia. Face recognition difficulties and autistic spectrum traits were reported more commonly in aphantasia. The Revised NEO Personality Inventory highlighted reduced extraversion in the aphantasia group and increased openness in the hyperphantasia group. Resting state fMRI revealed stronger connectivity between prefrontal cortices and the visual network among hyperphantasic than aphantasic participants. In an active fMRI paradigm, there was greater anterior parietal activation among hyperphantasic and control than aphantasic participants when comparing visualization of famous faces and places with perception. These behavioral and neural signatures of visual imagery vividness extremes validate and illuminate this significant but neglected dimension of individual difference.

## Introduction

Vision is our preeminent sense modality, deployed in the here and now. Most of us can also *imagine* the appearances of scenes and objects in their absence, enjoying an experience that is typically less vivid than “real seeing” but nonetheless has a distinctively visual feel: We refer to this as the experience of *visual imagery* (Pearson 2019). Visual imagery plays a prominent role, for most of us, in the subjective experience of daydreams, autobiographical recollection, future thinking, and nocturnal dreaming (Schacter and Addis 2007; Smallwood and Schooler 2015); it can influence perception (Pearson 2019), exerts a potent influence on emotions (Holmes and Mathews 2010), and has implicated in creativity and mental practice (Shepard 1988; Munzert and Lorey 2013). Yet the experience of imagery proves to be highly variable. We have recently described individuals with a lifelong lack of visual imagery, or “aphantasia,” and others whose imagery is “as vivid as real seeing” or “hyperphantasia” (Zeman et al. 2015; Zeman et al. 2020). Here, we report the first systematic study of the neuropsychological and neural signatures of aphantasia and hyperphantasia.

The 19th-century psychologist, Francis Galton, devised the earliest measure of the vividness of visual imagery (Galton 1880). Using his “breakfast table questionnaire” that invited participants to rate the “illumination, definition and coloring” of “your breakfast table as you sat down to it this morning,” Galton recognized that in some participants “the powers [of visualization] are zero” (p. 306). However, this phenomenon, the lifelong absence of a mind’s eye, has been almost entirely neglected until our recent descriptions (Zeman et al. 2015; Zeman et al. 2020). The only exception is a study suggesting that 2–3% of the undergraduate population are “lifelong non-imagers” (Faw 2009).

Previous work focusing on less extreme variations in imagery vividness indicates that they exert a particularly strong influence on autobiographical recollection (Palombo et al. 2018): Imagery vividness correlates positively with the fluency of autobiographical recall and the density of sensory details in autobiographical recollection (D'Argembeau and Van der Linden 2006; Vannucci et al. 2016). More vivid autobiographical memories, in turn, are associated with stronger activation in visual cortices at the time of recollection (Gilboa et al. 2004) and stronger resting state connectivity between the medial temporal and occipital lobes (Sheldon et al. 2016). Disorders affecting vision itself and visual imagery have been linked to autobiographical memory impairment (Rubin and Greenberg 1998; Greenberg and Knowlton 2014; Ahmed et al. 2018).

Beyond the domain of memory, studies focusing on the vividness of “object imagery” suggest that high—but not extreme—trait vividness scores are associated with enhanced performance on certain perceptual tasks, such as identifying degraded figures, and are overrepresented among artists; high “spatial imagery” scores, in contrast, are associated with enhanced performance in visuospatial tasks and are overrepresented among scientists (Blazhenkova and Kozhevnikov 2010). There is evidence for an association between lifelong face recognition difficulty—congenital prosopagnosia—and reduced imagery vividness (Gruter et al. 2009), and, conversely, an association between synesthesia and elevated levels of imagery vividness (Barnett and Newell 2008).

The neural basis of typical visual imagery has been explored extensively in recent decades, building on extensive preceding reflection and research on the cognitive nature of imagery (Kosslyn et al. 2006; Mackisack et al. 2016). The “visualization network” involves a combination of regions, including frontoparietal regions subserving executive/attentional control and the control of eye movements; areas involved in memory, with overlap with the default mode network; and “visual” regions including the fusiform and primary visual cortices (Winlove et al. 2018; Pearson 2019). The process of visualization has been conceptualized as “vision in reverse”: During imagery, the usual flow of information from the retina through the visual system to multimodal cortices involved in the control of behavior is reversed, exploiting the extensive feedback pathways that ordinarily play a more subtle and unobtrusive role in vision (Friston et al. 2012; Pearson 2019; Breedlove et al. 2020). The neural correlates of imagery *vividness*, specifically, are controversial: There is evidence for an inverse relationship between the strength and vividness of visual imagery and the surface area and excitability of the primary visual cortex, in contrast to positive relationships with the surface area of frontal regions (Bergmann et al. 2016; Keogh et al. 2020). Vividness correlates with the degree of activation of higher-order visual areas and regions of the limbic system or default mode network (Fulford et al. 2018). The neural basis of *extreme* imagery vividness has not been investigated previously, with the exception of case studies of patients whose imagery vividness has been perturbed by neurological or psychiatric disorder (Farah 1984; Bartolomeo 2008; Zeman et al. 2010).

Our description of “aphantasia” in 2015 attracted global publicity, resulting in email contact from >14 000 individuals, the majority describing lifelong aphantasia, but many also reporting its converse, lifelong hyperphantasia (Zeman et al. 2020). Data from a community-based biobank suggest a prevalence of 0.7% for “extreme aphantasia” and 2.5% for “extreme hyperphantasia,” defined as floor and ceiling scores on the Vividness of Visual Imagery Questionnaire (VVIQ) (Zeman et al. 2020). A questionnaire-based study among individuals with extreme imagery who contacted us spontaneously indicates that aphantasia is associated with reported difficulty in autobiographical memory and face recognition, while hyperphantasia is associated with an increased frequency of synesthesia (Zeman et al. 2020). Participants with aphantasia were significantly more likely to work in scientific or mathematical domains, while participants with hyperphantasia were more often employed in “creative” professions. Visual aphantasia is variably associated with the absence of imagery in other modalities, while visual imagery during dreaming is often preserved, suggesting a dissociation between wakeful and dreaming imagery. The frequency of aphantasia in first-degree relatives is elevated, hinting at a possible genetic contribution to imagery vividness. Although we did not enquire systematically about the presence of autistic spectrum disorder in our participants with extreme imagery, many people with aphantasia mentioned spontaneously that they fall on the autistic spectrum. Dawes et al. (2020) have recently reported broadly similar results from an independent questionnaire survey.

While these findings are suggestive, they rely on first-person report: Introspection can, of course, be fallible (Zeman et al. 2020). Differences in visual imagery vividness judgments could reflect differences in metacognition rather than true differences in vividness. There is therefore a need to triangulate these first-person findings with more objective measurements of both behavioral and neural correlates of visual imagery extremes. Such evidence has begun to accumulate in recent reports: People with aphantasia are unable to use imagery to modulate perception during binocular rivalry (Keogh and Pearson 2018); exhibit a reduced galvanic skin response to visually evocative narratives with a strong emotional impact on control participants (Wicken et al. 2019); show reduced precision of visual working memory (Jacobs et al. 2018); and recall fewer objects and colors from studied scenes than control participants (Bainbridge et al. 2021). These findings suggest that people reporting aphantasia have introspected a distinctive trait with measurable implications for psychological functioning.

We recruited participants with aphantasia, hyperphantasia, and average imagery vividness to investigate a range of hypotheses based on our questionnaire data. On the behavioral plane, we predicted that individuals with aphantasia would display impairments of autobiographical memory, future and atemporal imagination, and face recognition. On the neural plane, we predicted that there would be brain imaging signatures of visual imagery vividness extremes, which we investigated using a multimodal approach (structural MRI, task-based fMRI, resting state fMRI [rsfMRI], and diffusion tensor imaging). We suspected, more tentatively, that there might be differences in personality or traits relevant to autistic spectrum disorder between people with aphantasia and hyperphantasia. This suspicion is supported by some recent evidence (Dance et al. 2021). Finally, our previous work (Zeman et al. 2010) suggested that tests traditionally thought to be sensitive to differences in imagery vividness might discriminate poorly between these groups.

We note that the term “imagination” is ambiguous, as it can refer, among other things, to the ability to summon up an image of a specific item (“imagine an apple”) and to the richer ability to represent some possible or future scene or event (“imagine the next party you will go to”). We distinguish these two senses here by using the term “visualization” to refer to the former and “imagination” to refer to the latter.

## Materials and Methods

### Participants

Sixty-nine participants completed the study. Twenty-four participants had aphantasia, defined as having a Vividness of Visual Imagery Questionnaire (modified VVIQ; Marks 1973) score between 16–23/80 (Zeman et al. 2020), and twenty-five had hyperphantasia, defined as a VVIQ score between 75 and 80 (Zeman et al. 2020). Participants with aphantasia and hyperphantasia were recruited from a variety of sources (spontaneous email contacts to the research team, the Extend Biobank, and members of the University community). They reported that their imagery vividness had been a lifelong trait (i.e., not acquired). Twenty control participants with VVIQ scores in the midrange (55–60) were recruited from a local Biobank (http://exeter.crf.nihr.ac.uk/extend). The mean score VVIQ among the control participants (56.95) was similar to the mean score in our previous community-based control group (58.6). All participants (with aphantasia, hyperphantasia, and controls) satisfied the requirements that 1) they could attend in person on two occasions for testing and scanning; 2) they were otherwise healthy; and 3) they were right-handed. Seven participants (three with aphantasia, three with hyperphantasia, and one control) underwent neuropsychological testing but not MRI scanning for a variety of reasons (obesity, metallic implants, scanner failure, and logistical reasons) and were therefore excluded from the study. Ethical approval for this study was obtained from the University of Exeter Psychology Ethics Committee. As expected, there was a significant overall difference in the VVIQ score between groups, *F*(2,66) = 5245.53, *P* < 0.001, η^2^*_p_* = 0.994. Pairwise comparisons indicated that the VVIQ score for the aphantasia group (M = 16.92, SD = 1.47) was significantly lower than for both the control (M = 56.95, SD = 2.93) and hyperphantasia (M = 77.08, SD = 1.75) groups (both *P*s < 0.001), while the VVIQ score for the hyperphantasia group was significantly higher than for the control group (*P* < 0.001).

### Behavioral Methods

#### Neuropsychological Profile

Standard neuropsychological tests were used to investigate general intelligence (Weschler Abbreviated Scale of Intelligence; Wechsler 2011 or the Weschler Adult Intelligence Scale; Wechsler 2008) and executive functioning (Trails test and letter [FAS] and animal fluency, e.g., Steinberg et al. 2005). Depression and anxiety were assessed using the Hospital Anxiety and Depression Scale (Zigmond and Snaith 1983). The Autism Spectrum Quotient questionnaire (Baron-Cohen et al. 2001) and the NEO Five-Factor Inventory-3 (McCrae and Costa 2010) were also administered.

#### Anterograde Memory

Anterograde memory was measured using the Logical Memory Test (immediate and 30 min delayed recall as well as 30 min delayed recognition of a prose passage) (Wechsler 1997), the Rey–Osterrieth Complex Figure Test (copy and 30 min delayed recall) (Osterreith and Rey 1944), and the Warrington Recognition Memory Test (Warrington 1984) for word and face recognition.

#### Visual Imagery Tests

We used the Manikins test (Ratcliff 1979) to assess mental rotation. In this test, a manikin holding a black disk in one hand was either presented facing the participant or with its back to the participant and was standing either the right way up or upside down. Participants had to say which hand the black disk was in. There were 3 practice trials and 32 experimental trials.

In the curved segment test (van der Meulen et al. 2009), each letter in the alphabet was shown in lower case. Participants were asked to say whether the upper-case letter (not shown) corresponding to each lower-case letter has a curved line segment to it (e.g., “A” would not have a curved segment, while “P” would).

In the animal tails test (Behrmann et al. 1994), the names of 40 animals were presented (e.g., hedgehog and squirrel) and participants were asked to judge whether the animal had a short or a long tail relative to its body size. In all three tests, accuracy was the dependent measure.

#### Future and Atemporal Imagination

The future and atemporal imagination tasks were closely based on the procedures previously described by Hassabis et al. (2007). For the atemporal imagination task, participants were provided with three scenarios (1) imagine that you are sitting on a white sandy beach on a beautiful tropical bay; 2) imagine that you are standing in the main hall of a museum containing many exhibits; 3) imagine that you are standing in the middle of a bustling street market), read out by the experimenter. Participants were asked to imagine each scenario and describe it in as much detail as possible. Participants were told not to recount an actual memory or any part of one but to create something new. Participants continued until they came to a natural end. Extra details were then sought by the experimenter via general probes (e.g., can you see anything else in the scene?). The experimenter did not introduce any piece of information that had not already been described by the participant. After this, the participants rated their scenarios on a scale of 1 to 5 in terms of their sense of presence (1, “did not feel like I was there at all”; 5, “felt strongly like I was really there”) and perceived salience (1, “couldn’t really see anything”; 5, “extremely salient”).

In the future imagination task, participants were asked to imagine three events in the near future (1) possible Christmas event; 2) possible event over next weekend; 3) possible future meeting with a friend) that were specific in time and place, and realistic in nature. Participants were told that it should not be a past event transferred to the future. As in the atemporal imagination test, participants were asked to imagine the event and describe it in as much detail as possible. After the participants had finished their description, the experimenter provided general prompts to extract further information. At the end of the scenario, participants were again asked to rate on a scale from 1 to 5 their sense of presence and perceived salience.

Scoring for both tasks was conducted in the manner described by Hassabis et al. (Hassabis et al. 2007) with the exception that we did not include the spatial coherence index measure. The reason for this is that this self-report index asks participants specifically to describe the nature of the visual imagery they are experiencing (e.g., “It was a collection of separate images.”), which would be difficult for the aphantasia group to rate in the same way as the other groups. This resulted in a composite score out of 54 comprising three components. For the first component—*Content*—the scorer (K.K.), who was blind to the groups to which participants belonged, classified statements by the participant as belonging to one of the following four categories: spatial reference, entity presence, sensory description, or thought/emotion/action. Repetitions or irrelevant information was not scored. Following the original protocol (Hassabis et al. 2007), the score for the number of unique details in a category was capped at 7, making a total content score of 28. The second component was the *Participant ratings* detailed above (although the ratings were rescaled from 1–5 to 0–4, the overall measure was scored out of 8). The final component was the scorer’s own assessment of the extent to which the description evoked a detailed picture of the experience in their own mind’s eye (0, no picture at all; 10, vivid, extremely rich picture). Following the original protocol (Hassabis et al. 2007), this score was multiplied by 1.8 to give a measure scored out of 18. We averaged the composite score of the three scenarios for both the atemporal and future imagination tasks (One control participant was excluded from the future imagination task due to technical problems with the audio recording. For similar reasons, the average future imagination score for one of the hyperphantasia participants was based on two rather than three scenarios.). A second scorer (C.D.) analyzed a subset of the descriptions (32%) independently. Coefficients showed that the agreement between the scorers was high for both the atemporal (0.99) and future imagination (0.97) tasks.

#### Autobiographical Memory

The Autobiographical Interview (Levine et al. 2002) involved three phases: A recall stage where participants were asked to recollect as much information as they could about a unique, temporally restricted, event that they were personally involved in for both an event in the most recent 5 years (but not in the previous 3 months) and an event that was more than 5 years old (the remote time period); a general probe consisting of nonspecific questions (e.g., “can you provide more details?”); and a specific probe where participants underwent a semistructured interview in order to extract as much contextual detail as possible. The specific probe was administered after completing the free recall and general probe phases for each memory.

The memories were audiorecorded, transcribed, and scored using standard procedures (Levine et al. 2002). Specifically, narratives were segmented into details classified as “internal” or “external.” Internal details were episodic information specific to the selected event that can be subdivided into event, perceptual, time, place, and emotion/thought details. External details included information extrinsic to the event and consisted of semantic (factual information or extended events) and “other” (e.g., metacognitive statements, editorializing, and inferences) details. Contextual information that was not part of the chosen episode was also classified as external detail. Repetitions were scored but not included as either internal or external details (c.f., Milton et al. 2010). Additionally, qualitative ratings were assigned to each memory (Levine et al. 2002). The time, place, perceptual, and emotion/thought subcategories were rated on a scale from 0 (no information relating to that subcategory) to 3 (specific, rich detail pertaining to the subcategory). Episodic richness was scored on a 0–6 scale to account for its greater importance. A time integration measure (on a 0–3 scale) assessed the integration of the episode into a larger time scale. The ratings summed to 21. The interviews were all analyzed by one scorer (F.M.), for consistency, who was blind to the groups to which participants belonged. A second scorer (C.D.) analyzed a subset of the memories (32%) independently. Coefficients showed that the agreement between the scorers was high for the internal details (0.89), external details (0.92), and the qualitative ratings (0.95).

#### Face and Buildings Recognition

Participants completed the 20-item Prosopagnosia Index (PI20) questionnaire (Shah et al. 2015) to test for perceived face recognition difficulties. In addition, we developed a 15-item famous faces test, in which participants were presented with four similar faces and they had to pick out which was the famous face (e.g., Barack Obama and George Clooney). In the graded buildings test (Evans et al. 1995), participants were required to identify 30 buildings varying from easy (e.g., the Leaning Tower of Pisa) to hard (e.g., Sagrada Familia). Each building was scored out of 2 (1 mark for correct names, 1 for correct location).

Neuropsychological tests were administered in the same order to all participants during a single testing session, lasting approximately 3 h. If participants became fatigued during testing, they were encouraged to take a break, but this was seldom required. Questionnaires were completed prior to the scanning session. For logistical reasons, neuroimaging and neuropsychological testing were performed on the same day in 30 participants, on different days in 39 participants.

#### Correction for Multiple Comparisons

As there is no consensus on the appropriate correction for multiple comparisons in neuropsychological studies, we have given uncorrected probabilities but also indicate whether these survive a strict Bonferroni correction on the basis that we have performed 34 tests (0.05/34 = 0.0015). To avoid repetition, we indicate the findings that remain significant at the corrected threshold with a bold asterisk^*^.

### Neuroimaging Methods

Scanning was performed using a 1.5 T system (Intera, Philips, the Netherlands) at the Exeter University Magnetic Resonance Research Centre (Exeter, UK). fMRI was undertaken using a T2^*^-weighted single-shot echoplanar (EPI) scanning sequence (Repetition time [TR] = 3 s, Echo time [TE] = 50 ms, resolution 2.88 × 2.88 × 3.6 mm, 38 slices, 90° flip angle) and comprised two imaging runs, each of 346 dynamics, resulting in a scanning time of 17.5 min per run. Resting state data were acquired using a T2^*^-weighted single-shot EPI scanning sequence (TR = 3 s, TE = 50 ms, resolution 2.5 × 2.5 × 3.5 mm, 37 slices, 90° flip angle) with 160 repetitions and a total acquisition time of 8 min. Diffusion tensor imaging (DTI) (TR = 9470 ms, TE = 66 ms, resolution 2.3 × 2.3 × 3 mm, 48 slices) was undertaken with 32 diffusion directions with *b* values of 0 and 1000 s mm^−2^. High-resolution T1-weighted anatomical images, which covered the whole head and were centered approximately over the anterior commissure, were acquired with a resolution of 0.9 × 0.9 × 0.9 mm. The order of acquisition was as follows: survey scans, fMRI, resting state, structural, DTI.

#### Resting State fMRI

During the resting state protocol, participants were instructed to stay awake, keep their eyes open, and look at the projection screen on which was displayed a cross. They were further instructed to look in the general direction of the cross but not to fixate on it in a concentrated fashion. Resting state analysis was undertaken in the CONN software package (www.nitrc.org/projects/conn, RRID:SCR_009550). Initially, preprocessing was carried out on the raw data, consisting of realignment, normalization, outlier detection (ART-based scrubbing. Using the 95th percentile in normative data, a global signal z-value threshold of 3, and participant motion threshold of 0.5 mm), and smoothing (8 mm). Regions of interest (ROI) were then specified based on the FSL Harvard-Oxford atlas (Desikan et al. 2006) covering cortical and subcortical areas, the automated anatomical labeling atlas (AAL) (Tzourio-Mazoyer et al. 2002) for cerebellar regions, and the CONN list of commonly used networks (Whitfield-Gabrieli and Nieto-Castanon 2012). Denoising was subsequently undertaken to remove motion associated with physiological and artefactual effects via the use of linear regression (using global white matter signal, CSF signal, realignment parameters, and scrubbing parameters as confounds) and band-pass filtering. Based on previous findings (Winlove et al. 2018; Pearson 2019), analysis was then undertaken at a seed-to-voxel level using the following regions/networks as the initial seed: Frontoparietal lateral prefrontal cortex (LPFC) network, dorsal attention network, visual–occipital network, and the hippocampus. Significance was defined as occurring with an uncorrected *P* < 0.001 for the height threshold and *P* < 0.05 for the FDR corrected cluster size.

#### Task-Based fMRI

Prior to the scanning protocol, participants were shown a series of color images corresponding to famous places and faces and asked to identify them. From this larger set, a subset of 40 famous faces with neutral expressions presented from the front and 40 famous places, which the participants were familiar with and had correctly identified, were used for the subsequent fMRI protocol.

During each of two runs of the fMRI protocol, participants undertook a modified version of the task performed by MX (Zeman et al. 2010), which consisted of three different tasks, grouped into blocks and separated by a blank white screen displayed for between 0.5 and 2.5 s, with the exact length randomized from set to set. The task was undertaken within a darkened scanner room where the only illumination was provided via the projection of the task images that filled the full visual field of the participants in the direction they were facing. In the first “Perception” block, a famous place or face image was presented for 6 s, followed by a response screen for 2 s where participants were instructed to rate the pleasantness of the image they saw on a 1–3 scale (from least to most pleasant) via a three-button response pad, activated by their right (dominant) hand, having previously ensured all text was clearly readable by the participant within the scanner. Subsequently, a blank screen was shown for a randomized length between 0.5 and 2.5 s. Four sequential image/response/blank screen sets were presented per block. The following “Control” block (Stark and Squire 2001) consisted of a single-digit number shown for 2 s followed by a feedback screen for 2 s where participants were instructed to indicate whether the number was odd or even via the first two buttons of the three-button response pad. A blank screen was then shown for a randomized length between 0.5 and 2.5 s. Four trials were presented per block. In the final “Imagery” block, participants were shown a screen for 6 s instructing them to imagine a named person or place, corresponding to one of the images they had seen earlier in the protocol (the specific instruction was to “think about” the person or place). This was then followed by a feedback screen for 2 s where participants were instructed to rate the vividness of the visualization experience on a 1–3 scale (least to most vivid) via the three-button response pad, after which a blank screen was shown for a randomized length between 0.5 and 2.5 s. Four sequential image/feedback/blank screen sets were presented per block. The sequence of Perception–Control–Imagery blocks outlined above was repeated 10 times per imaging run, resulting in 40 cases of each stimuli type per run.

Feedback responses generated within the task in terms of visualization vividness and pleasantness of images were analyzed by one-way ANOVAs to examine differences between groups and by paired t-tests to assess differences within groups between the “face” and “place” tasks. All subsequent data analysis was undertaken using SPM12 software (www.fil.ion.ucl.ac.uk/spm). The data from the two separate fMRI runs were treated as separate sessions within the analysis, which consisted of images being slice time corrected, realigned, coregistered to the T1 structural images, normalized to the Montreal Neurological Institute template (MNI305), and smoothed using a Gaussian kernel of 8 mm full-width half-maximum. Following estimation using a general linear model, employing a hemodynamic response function, together with temporal and dispersion derivatives to model the blood oxygen level–dependent response and including six head movement parameters as regressors, statistical analysis was carried out to compare activation patterns associated with the Perception, Control, and Imagery conditions for each individual. Whole brain comparisons were then undertaken at a groupwise level with a combined statistical threshold of *P <* 0.001 and a threshold of 28 contiguous voxels (voxel size: 3 × 3 × 3 mm), which together produce an overall corrected threshold of *P* < 0.05. These values were estimated using AlphaSim as implemented in the REST toolbox (Version 1.8, Song et al. 2011). A smoothing estimate of 9.3, 9.1, 9.2 mm was used (this was a group-level estimate calculated in SPM12 using the group residuals from the general linear model, e.g., Kiebel et al. 1999).

#### Volumetric MRI

Differences between groups in sizes of brain substructures were assessed by undertaking voxel-based morphometry using the Dartel toolbox (Ashburner 2007) within SPM12 (the Wellcome Department of Cognitive Neurology, University College London). Data was segmented from individuals to generate gray and white matter images. Non-linear deformations of these images were established to ensure that these matched across all participants. Finally, data was normalised to MNI space. Statistical analysis was then undertaken via the use of a linear general model to examine systematic differences between the groups taking into account differences in total brain size between individuals. Significant changes were accepted at a *P* < 0.05 following family-wise error correction.

#### Diffusion Tensor Imaging

Voxelwise statistical analysis of the fractional anisotropy (FA) data was carried out using tract-based spatial statistics (TBSS, Smith et al. 2006), part of FSL (Smith et al. 2004). First, FA images were created by fitting a tensor model to the raw diffusion data using FDT, within the FSL toolbox, and then brain-extracted using BET (Smith 2002). All subjects’ FA data were then aligned into a common space using the nonlinear registration tool FNIRT (Andersson et al. 2007a, 2007b), which uses a b-spline representation of the registration warp field (Rueckert et al. 1999). A mean FA image was then created and thinned to create a mean FA skeleton that represents the centers of all tracts common to the group. Each subject’s aligned FA data were then projected onto this skeleton and the resulting data fed into voxelwise cross-subject statistics. Statistics were undertaken using the “Randomize” tool within FSL, employing the threshold-free cluster enhancement (TFCE) method, which generates cluster-based thresholding corrected for multiple comparisons, with significant changes accepted at a *P* < 0.05.

### Data-Sharing

Requests for data-sharing should be addressed to the authors and will be considered individually. We are keen to share data where possible, respecting the nature of the consent provided by our participants.

## Results

### Demographic and Behavioral Measures

The demographic, neuropsychological, and personality profiles of the aphantasia, control, and hyperphantasia groups are displayed in Table 1. There was no significant difference in age between the groups, *F*(2,66) = 0.12, *P* = 0.884, η^2^*_p_* = 0.004. There was also no difference in gender distribution across groups, χ^2^ (2,69) = 3.98, *P* = 0.136. However, IQ did differ significantly across the groups, *F*(2,66) = 5.18, *P* = 0.008, η^2^*_p_* = 0.136. Pairwise comparisons indicated that the aphantasia group had a significantly elevated IQ compared with the hyperphantasia group (*P* = 0.002), but there were no other statistically significant differences.

**Table 1:** Demographic, neuropsychological, and personality profile of the aphantasia, control, and hyperphantasia groups

	Aphantasia mean (SD)	Control mean (SD)	Hyperphantasia mean (SD)
Gender	14 male/10 female	11 male/9 female	8 male/17 female
Age, years	33.71 (11.25)	34.6 (12.78)	35.36 (11.10)
IQ	115.46 (12.54)	110.00 (11.41)	106.32 (4.51)
Trail A (s)	22.84 (9.01)	21.62 (5.79)	20.42 (6.01)
Trail B (s)	40.18 (9.45)	40.74 (9.65)	46.67 (16.74)
Letter fluency (words/min)	15.65 (4.80)	17.22 (3.35)	16.60 (4.60)
Animal fluency (words/min)	26.67 (5.70)	28.20 (5.59)	25.76 (5.43)
Autism quotient score (/50)	22.04 (7.99)	14.20 (6.24)	17.92 (10.40)
*Hospital Anxiety and Depression Scale scores (max score)*			
Anxiety (/21)	7.00 (4.67)	7.10 (2.83)	7.16 (5.01)
Depression (/21)	4.21 (2.98)	3.55 (3.10)	2.28 (2.82)
*Revised NEO PI (/25–75)*			
Neuroticism	48.96 (14.94)	51.45 (10.30)	48.96 (12.30)
Agreeableness	53.58 (12.03)	54.65 (11.45)	52.00 (10.47)
Extraversion	46.54 (11.62)	57.75 (11.80)	57.16 (13.82)
Openness to experience	53.21 (12.37)	57.05 (10.40)	65.16 (11.39)
Conscientiousness	42.13 (10.17)	49.5 (9.65)	47.96 (11.67)

There was no difference between groups in either Trails A, *F*(2,66) = 0.70, *P* = 0.499, η^2^*_p_* = 0.02, or Trails B, *F*(2,66) = 1.95, *P* = 0.151, η^2^*_p_* = 0.06. There were also no differences between groups for letter (FAS), *F*(2,66) = 0.73, *P* = 0.486, η^2^*_p_* = 0.02, and Animals, *F*(2,66) = 1.07, *P* = 0.347, η^2^*_p_* = 0.03, verbal fluency.

On the HADS anxiety measure, there was no overall difference between groups, *F*(2,66) = 0.01, *P* = 0.992, η^2^*_p_* < 0.001. For depression, there was also no overall difference, *F*(2,66) = 2.69, *P* = 0.076, η^2^*_p_* = 0.08.

For the Autism Quotient measure, there was a significant overall difference between groups, *F*(2,66) = 4.64, *P* = 0.013, η^2^*_p_* = 0.12. Pairwise comparisons revealed that the aphantasia group had a significantly higher score than the controls (*P* = 0.003). There was no statistical difference in scores between the hyperphantasia and control groups (*P* = 0.151).

Turning to the revised NEO Personality Inventory, there was no overall difference between groups for Neuroticism, *F*(2,66) = 0.27, *P* = 0.764, η^2^*_p_* < 0.01, or Agreeableness, *F*(2,66) = 0.32, *P* = 0.731, η^2^*_p_* < 0.01 (in both cases, all pairwise comparisons, *P* > 0.4). However, there was a significant overall difference in terms of Extraversion, *F*(2,66) = 5.93, *P* = 0.004, η^2^*_p_* = 0.15. Pairwise comparisons revealed that the aphantasia group scored significantly lower on this measure than both the control (*P* = 0.004) and hyperphantasia (*P* = 0.004) groups. There was no difference in Extraversion between the control and hyperphantasia groups (*P* = 0.876). There were also differences between groups on the Openness to Experience measure, *F*(2,66) = 6.92, *P* = 0.002, η^2^*_p_* = 0.17, with the hyperphantasia group scoring significantly higher on this measure than both the aphantasia (*P* < 0.001) and control (*P* = 0.021) groups. There was no difference between the aphantasia and control groups (*P* = 0.273).

### Anterograde Memory

The mean scores across groups for all the anterograde memory tests are shown in Table 2. There were no significant differences between groups for either the copy, *F*(2,66) = 0.82, *P* = 0.443, η^2^*_p_* = 0.02, or delayed recall, *F*(2,66) = 0.12, *P* = 0.294, η^2^*_p_* = 0.04, of the Rey–Osterrieth Complex Figure Test.

**Table 2:** Performance on the anterograde memory tests across groups

Anterograde memory scores (max score)	Aphantasia mean (SD)	Control mean (SD)	Hyperphantasia mean (SD)
Logical memory test			
Immediate recall (25)	11.08 (3.44)	13.45 (4.70)	13.80 (4.29)
Recall delay (25)	10.58 (3.60)	12.55 (4.86)	12.16 (3.37)
Recognition delay (15)	12.21 (1.74)	12.45 (2.01)	13.00 (1.29)
Rey–Osterrieth Complex Figure			
Copy (36)	35.33 (1.09)	35.70 (.92)	35.64 (1.08)
Delayed recall (36)	23.17 (5.28)	24.95 (3.99)	25.08 (4.51)
Warrington Recognition Test			
Words (50)	49.04 (1.40)	49.10 (1.17)	49.08 (1.08)
Faces (50)	42.79 (4.46)	43.35 (4.85)	44.48 (2.58)

For the immediate recall of the logical memory test, there was no statistically significant difference between groups, *F*(2,66) = 3.03, *P* = 0.055, η^2^*_p_* = 0.08. There were no overall differences between groups for either the 30 min delayed recall, *F*(2,66) = 1.61, *P* = 0.209, η^2^*_p_* = 0.05, or recognition, *F*(2,66) = 1.42, *P* = 0.249, η^2^*_p_* = 0.04, tests. There were also no statistical differences between groups for the Warrington Recognition Words, *F*(2,66) = 0.01, *P* = 0.987, η^2^*_p_* < 0.001, and Faces, *F*(2,66) = 1.12, *P* = 0.332, η^2^*_p_* = 0.03, tests.

### Visual Imagery Tests

The mean scores across groups for each of the visual imagery tests are displayed in Table 3. There was no overall difference between groups for the Manikins test, *F*(2,66) = 1.85, *P* = 0.165, η^2^*_p_* = 0.05, or for the curved segments test, *F*(2,66) = 0.24, *P* = 0.789, η^2^*_p_* = 0.01. For the animal tails test, the overall difference between conditions narrowly missed conventional statistical significance, *F*(2,66) = 3.07, *P* = 0.053, η^2^*_p_* = 0.09.

**Table 3:** Performance on the visual imagery tests across groups

Visual imagery test (max score)	Aphantasia mean (SD)	Control mean (SD)	Hyperphantasia mean (SD)
Manikins test (32)	30.42 (3.16)	29.75 (2.47)	31.20 (1.78)
Curved segments test (26)	25.63 (0.92)	25.65 (0.67)	25.76 (0.52)
Animal tails test (40)	32.92 (3.65)	34.05 (2.74)	35.00 (2.27)

### Future and Atemporal Imagination

**Figure 1:**
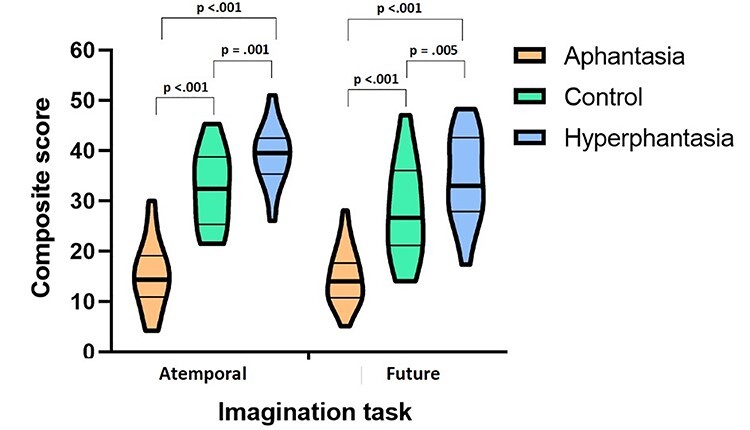
Mean composite score (maximum = 54) for each group in the atemporal and future imagination tasks. The thick line within the violin plots display the median score, the thinner lines reflect the 25th and 75th percentiles. The top and bottom of the violin plots illustrate the lowest and highest scores in the sample. The plots were created using Prism GraphPad Prism v. 9 (https://www.graphpad.com/scientific-software/prism/).

The mean scores across conditions for the atemporal and future imagination tasks are displayed in Figure 1. For the atemporal imagination task, there was a significant overall difference between groups, *F*(2,66) = 86.88, *P* < 0.001^*^, η^2^*_p_* = 0.73, with the aphantasia group scoring significantly lower than the control and hyperphantasia groups (both *P*s < 0.001), while the hyperphantasia group scored significantly higher than the control group (*P* = 0.001).

There was also a significant overall difference between groups for the future imagination task, *F*(2,65) = 41.21, *P* < 0.001^*^, η^2^*_p_* = 0.56, with pairwise comparisons revealing that the aphantasia group had a significantly lower composite score than the control and hyperphantasia groups (both *P*s < 0.001). Furthermore, the hyperphantasia group had a significantly higher score than the control group (*P* = 0.005).

Whilst there was no clear departure from normality for any of the conditions, as a safety check we run the same analyses using the non-parametric Kruskal-Wallis test with follow-up pairwise comparisons conducted using Mann-Whitney U tests. These analyses led to exactly the same conclusions as the results reported above.

### Autobiographical Memory

**Figure 2:**
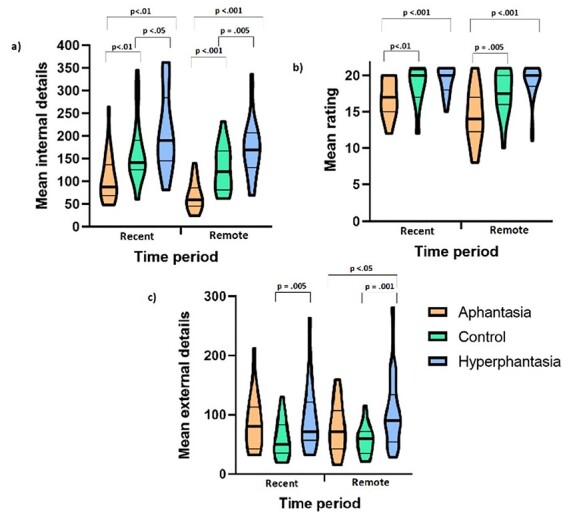
Violin plots showing: (*a*) Mean number of internal details recalled for each time period; (*b*) mean rating (out of 21) for each time period; (*c*) mean number of external details recalled for each time period. The thick line within the violin plots displays the median score; the thinner lines reflect the 25th and 75th percentiles. The top and bottom of the violin plots illustrate the lowest and highest scores in the sample. The plots were created using Prism GraphPad Prism v. 9 (https://www.graphpad.com/scientific-software/prism/).

Mean scores for the internal and external details together with the qualitative ratings are shown in Figure 2. The mean number of internal details for the recent time period was analyzed using a one-way ANOVA and revealed a significant difference across groups, *F*(2,66) = 13.67, *P* < 0.001^*^, η^2^*_p_* = 0.293. Pairwise comparisons revealed that the aphantasia group produced significantly fewer internal details than both the control and hyperphantasia groups (both *P*s < 0.01). In addition, the hyperphantasia group generated significantly more internal details than the control group (*P* = 0.041). There was also a significant effect for the remote time period, *F*(2,66) = 27.74, *P* < 0.001^*^, η^2^*_p_* = 0.457. Pairwise comparisons again showed that the aphantasia group recalled significantly fewer internal details than both the control and hyperphantasia groups (both *P*s < 0.001), while the hyperphantasia group produced more internal details than the control group (*P* < 0.004).

Analysis of the qualitative rating for the recent time period also yielded a significant overall difference between groups, *F*(2,66) = 7.19, *P* < 0.002, η^2^*_p_* = 0.30, with the aphantasia group having a significantly lower mean score than both the control (*P* = 0.018) and hyperphantasia (*P* < 0.001) groups. There was no statistical difference between the control and hyperphantasia groups (*P* = 0.273) although the hyperphantasia group had a numerically higher average. A similar pattern emerged for the remote time period—there was a significant overall effect, *F*(2,66) = 13.62, *P* < 0.001^*^, η^2^*_p_* = 0.292 with the aphantasia group having a lower score than the control (*P* = 0.002) and hyperphantasia groups (*P* < 0.001). There was no significant difference between the control and hyperphantasia groups (*P* = 0.119).

For external details, there was a significant overall difference between groups for the recent time period, *F*(2,66) = 3.51, *P* = 0.035, η^2^*_p_* = 0.096, with the hyperphantasia group producing significantly more details than the control group (*P* = 0.002). The other comparisons were not statistically different (*P*s > 0.05). There was also a significant difference between groups for the remote time period, *F*(2,66) = 6.02, *P* = 0.004, η^2^*_p_* = 0.154. The hyperphantasia group recalled more external details than both the aphantasia (*P* = 0.044) and control (*P* = 0.001) groups. There was no statistical difference between the aphantasia and control group (*P* = 0.147).

For the autobiographical memory data, the data for some of the groups was of questionable normality. We again ran additional analyses using the Kruskal-Wallis test, using Mann-Whitney U tests for follow-up pairwise comparisons as a safety check. These analyses produced the identical pattern of results as presented above, with the exception that for the external details recent time period, the pairwise comparison revealed that the aphantasia group had a higher mean score than the control group (*P* = .040).

Finally, we calculated the ratio of internal:total details (c.f., Levine et al. 2002). These ratios for both remote and recent memories can be seen in Table 4. For the recent period, there was a significant overall difference between groups, *F*(2,69) = 18.97, *P* < 0.001^*^, η^2^*_p_* = 0.365. Pairwise comparisons revealed that the aphantasia group had a significantly lower ratio than both the control and hyperphantasia groups (both *P*s < 0.001). There was no statistical difference between the hyperphantasia and control groups (*P* = 0.154). For the remote time period, there was also a significant overall difference between groups, *F*(2,66) = 23.50, *P* < 0.001^*^, η^2^*_p_* = 0.416. The aphantasia group again had a significantly lower ratio than the control and hyperphantasia groups (*P* < 0.001), but there was no statistically significant difference between the control and hyperphantasia groups (*P* = 0.095).

**Table 4:** Internal to total ratios for the recent and remote time periods

	Recent		Remote	
	M	SD	M	SD
Aphantasia	0.56	0.12	0.48	0.13
Hyperphantasia	0.70	0.10	0.64	0.11
Control	0.74	0.08	0.69	0.074

### Face and Buildings Recognition

The mean scores for Prosopagnosia Index (PI) (Shah et al. 2015), the famous faces recognition test, and the graded buildings test are presented in Table 4. There was a significant overall difference between groups for the PI, *F*(2,66) = 12.83, *P* < 0.001^*^, η^2^*_p_* = 0.28, with the aphantasia group reporting their face recognition ability as significantly worse than both the control (*P* = 0.001) and hyperphantasia groups (*P* < 0.001). There was no difference in mean score between the control and hyperphantasia groups (*P* = 0.21).

In contrast to the results for the PI, there was no overall statistical difference between groups for the 15-item famous faces recognition test, *F*(2,66) = 0.67, *P* = 0.516, η^2^*_p_* = 0.02. The famous buildings test, though, did yield a significant overall difference between groups, *F*(2,66) = 3.43, *P* = 0.038, η^2^*_p_* = 0.09, with the aphantasia group performing significantly worse than the hyperphantasia group (*P* = 0.013). There were no other statistical differences (*P*s > 0.5).

### Brain Imaging Results

#### Resting State fMRI

**Figure 3:**
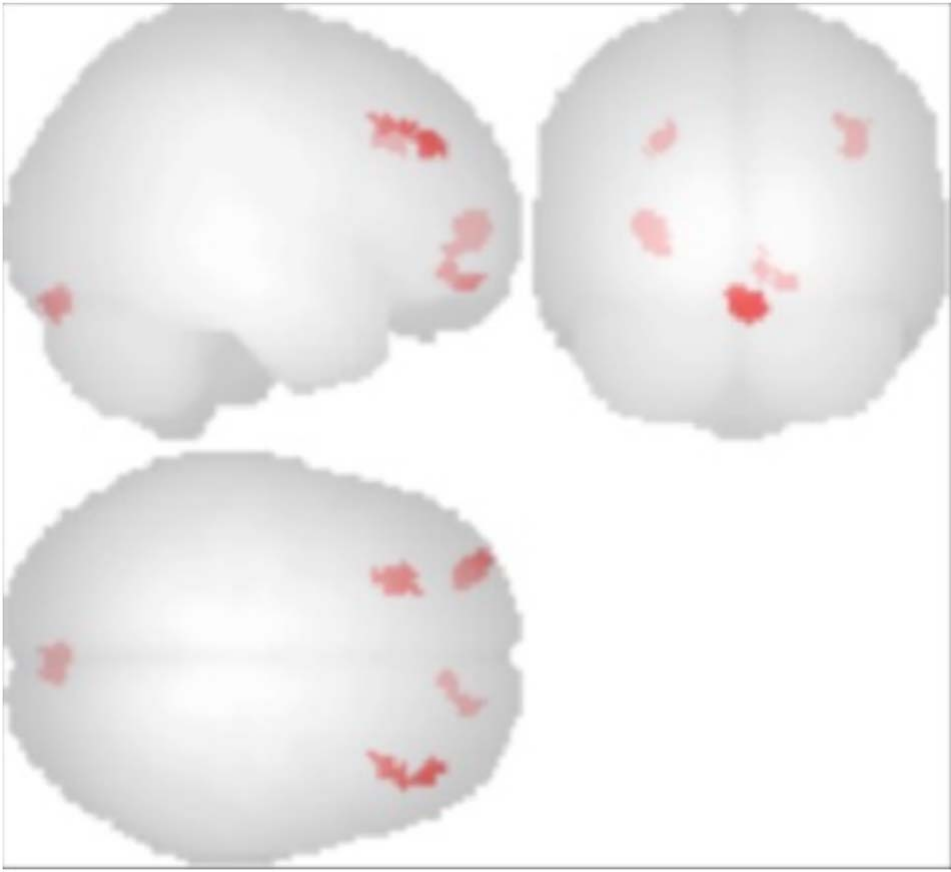
Seed to voxel connectivity analysis using the visual–occipital network as initial seed displaying areas of greater connectivity for hyperphantasic than aphantasic participants.

rsfMRI revealed stronger connectivity between the visual–occipital network and several prefrontal regions (BAs 9, 10, 11) in the hyperphantasic group when compared with the aphantasic group (see Table 5 and Fig. 3). The left hippocampus was more strongly connected to the brain stem in the hyperphantasic group than the aphantasic group. Stronger resting state connectivity was detected in the aphantasic group than in the hyperphantasic group between the left hippocampus and a region of the anterior cingulate (left BA24); the left dorsal attention network and the middle frontal gyrus; the left frontoparietal control network and the left orbitofrontal cortex (left BA11). Stronger connectivity was also found for the aphantasic group than the control group between the left hippocampus and left BA10; the left dorsal attention network and the right BA8. No significant increase in connectivity was determined for the control group relative to either the aphantasic or hyperphantasic groups or for the hyperphantasic group relative to the control group for any of the regions examined.

**Table 5:** Mean scores on the prosopagnosia index questionnaire and the famous face recognition and graded buildings tests across groups

	Aphantasia mean (SD)	Control mean (SD)	Hyperphantasia mean (SD)
Prosopagnosia Index (100)	55.71 (13.23)	43.30 (14.24)	38.72 (8.480)
Famous face recognition (15)	14.21 (1.22)	14.40 (1.00)	14.56 (9.96)
Graded buildings (60)	44.54 (7.37)	46.00 (7.46)	49.72 (6.59)

#### Task-Based fMRI

In terms of participant feedback recorded during the fMRI task, there were significant differences (*P* < 0.001) between the groups in the vividness of the visualization experience (Mean ± SD: Aphantasic 1.08 ± 0.25, Control 2.39 ± 0.31, Hyperphantasic 2.81 ± 0.23). However, none of the groups reported a significant difference between the face and place tasks in terms of visualization vividness (*P* > 0.05). There was no significant difference (*P* > 0.05) between groups for reported pleasantness of presented images. However, there was a significantly higher pleasantness rating for “places” compared with “faces” (*P* < 0.001) for all three groups.

**Figure 4:**
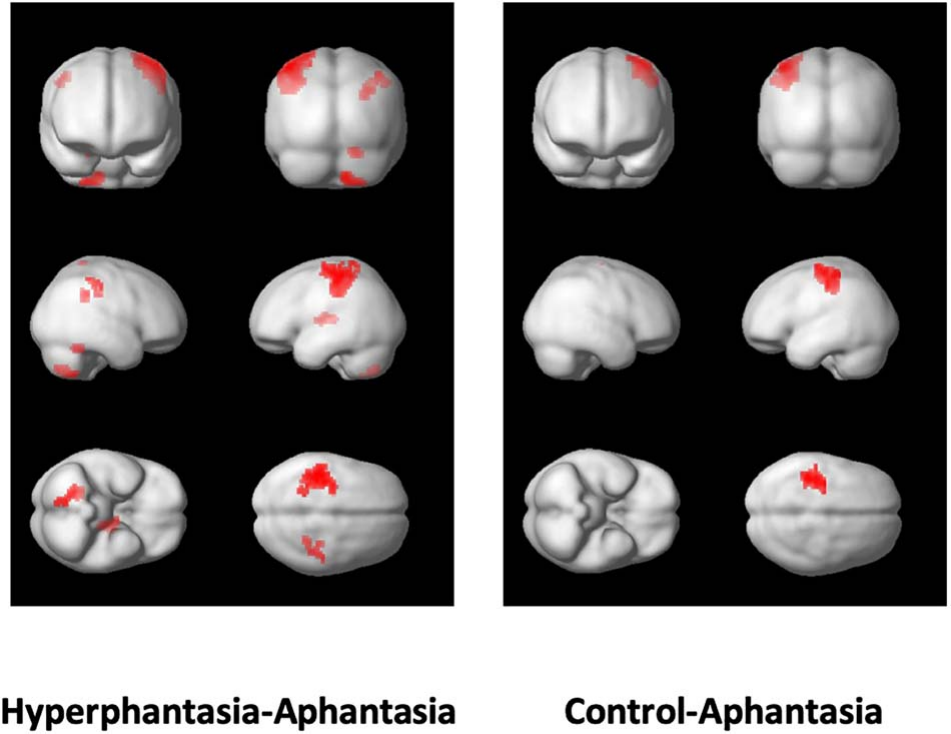
Areas more strongly activated in the hyperphantasic and control groups than the aphantasic group (left and right columns, respectively) in a subtraction of the perception from the visualization conditions.

In terms of brain activation, there were no significant group differences when comparing perception and visualization with the control condition (see Supplementary Figs 1–5*a*,b). However, hyperphantasic participants activated a left anterior parietal region in the precentral gyrus (BAs 3 and 4), extending into BA 40, to a greater degree than aphantasic participants, in a subtraction of perception from visualization (see Table 6 and Fig. 4). A smaller, symmetrically placed area was more strongly activated in the right hemisphere. Two regions of the cerebellum, within the posterior and anterior lobes, and an area of the thalamus, were also more strongly activated in this comparison in the hyperphantasic group. In a similar comparison between aphantasic and control participants, the same left anterior parietal region was more strongly activated in the control sample. No difference was identified between the control and hyperphantasic groups in this comparison (Table 7).

**Table 6:** Resting state connectivity differences between aphantasic and hyperphantasic groups

Network	Comparison	MNI coordinates of center of cluster	Anatomical region
Visual–occipital	Hyper > Aphan	−28, +56, +2	Left BA10
		+2, −92, −24	Cerebellum
		+40, +32, +38	Right BA9
		−24, +32, +36	Left BA9
		+8, +46, −12	Right BA11
Hippocampus-left	Hyper > Aphan	−2, −24, −54	Brain stem
Frontal parietal-left	Aphan > Hyper	−2, +56, −24	Left BA11
Left dorsal attention IPS (intraparietal sulcus)	Aphan > Hyper	−32, +22, +38	Middle frontal gyrus
Hippocampus-left	Aphan > Hyper	−10, −2, +40	Left BA24
Left dorsal attention IPS	Aphan > Control	+6, +36, +56	Right BA8
Hippocampus-left	Aphan > Control	−42, +40, +22	Left BA10

*Note:* The table identifies networks more strongly connected in the hyperphantasic or aphantasic group and the targets of differential connectivity

**Table 7:** Task-based fMRI: areas more strongly activated in hyperphantasic and control than aphantasic participants in a comparison of visualization with perception

Group comparison	Task comparison	Cluster center coordinates (MNI space)	Cluster center region	Cluster size (number of voxels)	*Z* Scores
Hyperphantasia > Aphantasia	Imagination-perception	−45 −22 62	Left anterior parietal	411	5.25
		−9 −7 5	Thalamus	56	4.45
		18 –61 −55	Cerebellum (anterior)	56	4.43
		33 –28 35	Right anterior parietal	45	4.22
		21 −52 −25	Cerebellum (posterior)	34	4.83
Control > Aphantasia	Imagination-perception	−39 −31 50	Left anterior parietal	225	5.52

#### Volumetric MRI

No significant differences in brain region volumes were found between the three groups.

#### Diffusion Tensor Imaging

No significant differences in fractional anisotropy were detected between groups for any brain regions.

## Discussion

Our study is the first wide-ranging exploration of the neuropsychological and neural associations of aphantasia and hyperphantasia. In keeping with their subjective reports (Dawes et al. 2020; Zeman et al. 2020), participants from these groups are distinguished by their scores on measures of autobiographical memory, future and atemporal imagination, and face recognition, but not by standard measures of anterograde memory. rsfMRI reveals stronger connectivity between the posterior visual network and prefrontal regions among people with hyperphantasia than those with aphantasia. In a comparison of visualization to perception, we found higher levels of brain activation centered on the anterior parietal lobe among people with hyperphantasia and control participants than in those with aphantasia. Aphantasia is associated with autistic spectrum traits and introversion, hyperphantasia with “openness.” Classic “visual imagery tasks” discriminate poorly between people with aphantasia and hyperphantasia. We discuss each of these findings in turn.

We predicted that aphantasia would be associated with relatively impoverished autobiographical memory, while hyperphantasia would be associated with enriched autobiographical memory, on the basis of our previous questionnaire data (Zeman et al. 2020). Despite a modest but statistically significant group difference in IQ in favor of the aphantasic group and essentially equivalent performance in the two groups on standard measures of anterograde recall and recognition memory for visual and verbal material, this prediction was strongly confirmed. Given the evidence that autobiographical memory performance is related to performance on measures of atemporal and future-directed imagination, and anecdotal evidence from some of our participants of difficulty with future thinking, we predicted that there would be similar differences in this domain. This prediction was also confirmed.

Autobiographical recollection, like the imagination tasks used in this study, requires participants to project themselves into mentally constructed scenes, engaging abilities that have been variously described in terms of “autonoesis” (Tulving 1985), scene-construction (Hassabis and Maguire 2007), self-projection (Buckner and Carroll 2007), and constructive episodic simulation (Schacter and Addis 2020). Such tasks make different demands to laboratory measures of recall and recognition for recently encountered material, helping to explain the dissociation evident here between performance on these two forms of memory test (Gilboa 2004; Palombo et al. 2018). The markedly contrasting performance seen in these tasks among participants at the extremes of the visual imagery vividness spectrum is in keeping with the more modest correlations between imagery vividness and the richness of autobiographical memory revealed in samples with typical imagery vividness (Vannucci et al. 2016; Palombo et al. 2018).

Why should extreme variations in imagery vividness be associated with such marked variations in autobiographical recollection and imagination? Both recollection and imagination typically involve evocation of sensory details—a “reexperiencing,” constrained by a past episode in the case of autobiographical memory, more autonomous in the cause of imagination (Palombo et al. 2018; Schacter and Addis 2020). The absence of the ability to evoke sensory details in this way, whether in the visual modality alone or across all sensory modalities (Dawes et al. 2020; Zeman et al. 2020), might be expected to have a profound impact on these cognitive processes. Clinical reports linking cortical visual impairment to autobiographical memory deficits (Rubin and Greenberg 1998; Gardini et al. 2011; Ahmed et al. 2018), taken together with evidence for visual memory impairment in the recently described Syndrome of Severely Deficient Autobiographical Memory (Palombo et al. 2015), are broadly consistent with our results. We consider the neural basis for this key finding below.

We predicted that we would find evidence of face recognition difficulty among participants with aphantasia, but not hyperphantasia, on the basis of subjective reports (Zeman et al. 2020). The significant elevation of PI scores among aphantasic participants supports this prediction. Although it is in keeping with an independent observation of an association between prosopagnosia and low imagery vividness scores (Gruter et al. 2009), we regard this finding as tentative. The PI is a self-report measure, albeit a validated one, and it could be that weak facial imagery has a metacognitive effect, reducing the confidence of people with aphantasia in their ability to recognize faces. Our measure of famous face recognition showed a ceiling effect and was therefore not an adequate objective measure. The contrast between the performance of the aphantasic and hyperphantasic groups on the Famous Building Test could reflect a perceptual or semantic effect and, like face recognition ability itself, requires further exploration.

Given that people with lifelong aphantasia do not describe major perceptual difficulties yet are unable to summon images to the mind’s eye voluntarily, reduction in connectivity between relevant cognitive control systems and visual cortices offers a plausible neural mechanism. The underlying thought here is that reverse activation of visual cortices in the service of visual imagery is likely to place stronger demands on reverse connections between frontoparietal executive regions and visual cortices than does visual perception (Dijkstra et al. 2019). This idea is strengthened by the observation that many people with aphantasia experience visual dreams (Dawes et al. 2020; Zeman et al. 2020), suggesting that they are capable of experiencing imagery when the requirement for voluntary imagery generation is removed. Our finding of reduced resting state connectivity between several areas of prefrontal cortex and the visual–occipital network among people with aphantasia, compared with those with hyperphantasia, and to control participants, supports an explanation along these lines. Comparable differences in connectivity have been reported in relation to autobiographical memory: Stronger resting state connectivity between the medial temporal lobes and visual cortices was seen in individuals reporting a more “episodic” style of autobiographical recollection, involving greater event detail, while individuals with a more factual style of recollection showed stronger connectivity between the medial temporal lobes and inferior and middle prefrontal regions (Sheldon et al. 2016). In a related finding, among patients with Parkinson’s disease, there is a stronger association between mind-wandering frequency and coupling between the default mode network and visual areas among patients with hallucinations as a manifestation of their disorder than among those who do not hallucinate (Walpola et al. 2020). Our observation of stronger resting state coupling between both lateral and medial prefrontal regions and visual cortices among participants with hyperphantasia than among participants with aphantasia is potentially relevant both to the difference in their subjective experience of imagery and to the differences in performance we report on measures of autobiographical memory and imagination.

Using our functional imaging paradigm, we did not find any difference between aphantasic and hyperphantasic groups in the most straightforward comparison of visualization versus control conditions in these two groups, in conflict with our initial hypothesis. However, we observed an activation difference in the anterior parietal cortex between hyperphantasic and control participants on the one hand and aphantasic participants on the other, in a comparison of visualization and perception. The anterior parietal area concerned, which was bilateral in the hyperphantasic/aphantasic comparison but larger in the left than right hemisphere, is close, but slightly anterior, to the area of maximum activation by visual imagery tasks in a recent meta-analysis (Winlove et al. 2018). We hypothesize that this activation difference reflects weaker deployment of visual attention within the parietal lobe during attempted visual imagery in our aphantasic participants than in hyperphantasic and control participants.

A number of aphantasic participants in our questionnaire study (Zeman et al. 2020) had mentioned spontaneously that they were on the autistic spectrum. Given that deficits in imagination, broadly construed, have been among the defining features of autistic spectrum disorder, this association appeared plausible. It is consistent with the observation from our questionnaire study of an association between aphantasia and mathematical and scientific occupations (Zeman et al. 2020), as these are associated, at a group level, with autistic traits (Baron-Cohen et al. 2001). The current study provides tentative support for a link between aphantasia and autistic spectrum features, with elevated Autism Spectrum Quotient questionnaire scores among aphantasic participants in comparison to the control group. This observation may relate to the rsfMRI findings discussed above, as autism has been associated with reduced long-range brain connectivity (Lai et al. 2014).

The association revealed by the NEO-FFI of aphantasia with reduced extraversion is in keeping with its association with elevated AQ scores, as introversion is itself associated with elevated AQ scores (Wakabayashi et al. 2006). In contrast, we found that hyperphantasia was associated with increased levels of openness. This construct is more fully described as reflecting “an openness to new experiences, broad interests, and an active imagination, and a likelihood of experiencing both positive and negative emotions more keenly than most people.” It is an intuitively plausible personality correlate for high levels of imagery vividness and has been linked to efficient information processing with the default mode network (Beaty et al. 2016) and, tentatively, to variation in the dopamine D4 receptor gene (*DRD4*) and the catechol-*O*-methyltransferase gene (*COMT*) (Deyoung et al. 2011).

We included three “standard” imagery tests as they have often been used as measures of imagery, despite doubts that they would be revealing in this context. The first two tests, the Manikins test and curved segments test, showed ceiling effects in our study, with scores over 90% correct in all three groups on the former and over 98% correct on the latter. They have other shortcomings in the context of visual imagery vividness extremes, as they primarily assess “spatial imagery,” which may be preserved in aphantasia (Keogh and Pearson 2018; Bainbridge et al. 2020; Dawes et al. 2020), rather than “object imagery,” which is likely to be the main locus of impairment. The animal tails test is, in contrast, directed to object imagery (Farah et al. 1988), but our previously described patient MX, with acquired aphantasia, scored normally on this test on the basis of “knowledge” rather than visualization (Zeman et al. 2010). The reduction of scores in the aphantasic group in the current study suggests that conscious visual imagery supports performance to some degree, although their 82% correct average score indicates, in keeping with the result in MX’s case, that other sources of knowledge make a contribution. More generally, these findings show that results of “imagery tests” must be interpreted cautiously, in the light of participants’ reports of their experience, as they are by no means pure “measures of visual imagery”: Other sources of knowledge, both general semantic knowledge and extravisual but modality-specific information, for example, kinesthetic imagery, can influence performance. Moreover, visual imagery, itself, is heterogeneous, incorporating “object” and “spatial” imagery, which appear likely to have distinct behavioral associations and neural bases (Blazhenkova and Kozhevnikov 2010; Kozhevnikov and Blazhenkova 2013).

Our study has a number of limitations. First, given the complexity of the neural network subserving visualization, and variability in the associated features of aphantasia (Zeman et al. 2020), it is likely that aphantasia is heterogeneous, with several subtypes: For example, we suspect that aphantasia in association with prosopagnosia will turn out to be distinct from aphantasia occurring in its absence. Hyperphantasia, also, is likely to be heterogeneous. In this study, we have not attempted to tease apart possible subtypes. We were concerned that resulting “noise” might obscure any neuropsychological and neural associations of extreme imagery. Our findings suggest, however, that there are at least some consistent differences between people at the two imagery vividness extremes. Second, anecdotal, and some experimental, evidence (Morina et al. 2013) indicate that imagery extremes are likely to have affective associations. We have not explored these in this study: This topic will be a fruitful area for further research (Holmes and Mathews 2010; Palombo and Levine 2018; Dawes et al. 2020). Third, while we found the classical “imagery tests” relatively insensitive to visual imagery vividness extremes, as discussed above, the measurement of response times, which we did not attempt, might have been more informative. Fourth, the IQ discrepancy between our aphantasic and hyperphantasic groups introduces a complexity into the interpretation of our results. However, as the discrepancy favored the aphantasic group, in which we found that autobiographical memory and imagination scores were *reduced*, if anything, differences between the groups in these respects are likely to have been *underestimated*. Fifth, we did not monitor eye movements during MRI scanning. This has not been undertaken, as a rule, in functional imaging studies of visual imagery, but future studies should ideally include this control, in case systematic group differences in eye movements may be influencing brain imaging findings. Sixth, in future studies, it would be desirable to include a control condition, such as picture description, to probe the possibility that the reduced scores on measure of autobiographical memory and imagination in the aphantasic group are related to a pervasive difference in narrative production. Finally, our use of a relatively low-powered scanner (1.5 T) and whole brain analyses makes it likely that we have detected only a minority of the structural and functional differences between the brains of people lying at the two visual imagery vividness extremes. Specifically, the current study has not applied a region of interest analysis to address the question of whether there are differences in the area of early visual cortices between participants at the two extremes of the vividness spectrum, as predicted by the findings of Bergmann et al. (2016). We plan to address this question in future work.

In conclusion, aphantasia and hyperphantasia are recently described extremes of visual imagery vividness, occurring in around 1% and 3% of the population, respectively. Spontaneous self-report and questionnaire data from several thousand participants have suggested that these imagery vividness extremes have a range of psychological and occupational associations (Dawes et al. 2020; Zeman et al. 2020). Here, we have shown that aphantasia is not associated with a reduction in performance on standard declarative memory tests. However, in keeping with self-report data (Zeman et al. 2020), it is associated with a marked reduction in performance on exacting measures of autobiographical memory and temporal and atemporal “imagination.” Also, in keeping with the self-report data, there are tentative associations between aphantasia and both face recognition difficulty and autistic spectrum traits. Hyperphantasia is associated with the personality trait of openness, while aphantasia is associated with a reduction in extraversion. rsfMRI reveals stronger functional connectivity between prefrontal regions and the visual–occipital network among hyperphantasic than aphantasic participants. Task-based fMRI identifies an anterior parietal region distinguishing the aphantasic sample from the control and hyperphantasic groups. Further work is required to explore the cognitive and behavioral advantages and disadvantages of what we suspect to be two key, contrasting, modes of human information processing, highlighted by the comparison of hyperphantasia with aphantasia: One more episodic and sensorily-rich, the other more semantic and factual. We anticipate that imagery extremes will have affective associations, which we have not studied here. A broader range of approaches to behavioral assessment and the use of more powerful imaging modalities have the potential to shed further light on this fundamental contrast.

## Notes

We are very grateful to the participants in this study. We thank Holly Wilson, Rachel Johnson, Amy Lewins, Kate Mitchell, and Sarah Mathew for help in collecting the brain imaging data, and Mada Radu for help with collecting behavioral data and data curation. We would also like to thank The NIHR Exeter Clinical Research Facility and the EXTEND project (https://exetercrfnihr.org/about/exeter-10000/) for assisting us with the recruitment of participants for this study. *Conflict of Interest*:

The authors declare no competing interests.

## Funding

United Kingdom Arts and Humanities Research Council Science in Culture Innovation Award: The Eye’s Mind—a study of the neural basis of visual imagination and its role in culture (AH/M002756/1); Follow-on Funding Award: Extreme Imagination in Mind, Brain and Culture (AH/R004684/1).

## Author Contributions

Conceptualization: Zeman; Data curation: Dance, Gaddum, Heuerman-Williamson, Jones, Zeman; Formal analysis: Fulford, Milton; Funding acquisition: Mackisack, Winlove, Zeman; Investigation: Dance, Fulford, Gaddum, Jones, Knight, Milton, Heuerman-Williamson, Jones; Methodology: Dance, Fulford, Gaddum, Milton, Heuerman-Williamson, Jones, Winlove, Zeman; Project administration: Winlove, Zeman; Resources: Zeman; Supervision: Fulford, Milton, Winlove, Zeman; Validation: Fulford, Milton, Zeman; Visualization: Fulford, Milton; Writing—original draft: Zeman; Writing—review and editing: all authors.

## Supplementary Material

Behavioral_and_Neural_Signatures_supplementary_data_for_submission_15_4_21_tgab035
