# Supplementary material for: Behavioral and Neural Signatures of Visual Imagery Vividness Extremes: Aphantasia versus Hyperphantasia
Source: Cereb Cortex Commun. 2021 May 5;2(2):tgab035. doi: 10.1093/texcom/tgab035 (PMC8186241; doi:10.1093/texcom/tgab035)
Supplement: Behavioral_and_Neural_Signatures_supplementary_data_for_submission_15_4_21_tgab035 [file behavioral_and_neural_signatures_supplementary_data_for_submission_15_4_21_tgab035.docx]

**Behavioral and neural signatures of visual imagery vividness extremes: supplementary data**

In all cases whole brain comparisons were undertaken at a groupwise level with a combined statistical threshold of *p < .*001 and a threshold of 28 contiguous voxels (voxel size: 3 x 3 x 3 mm), which together produce an overall corrected threshold of *p < .*05. Regions meeting this statistical threshold with greater activation in the first task named relative to the second are shown in red in the figures below.

**Figure 1**

**Perception task v control task: all participants combined (n=69)**


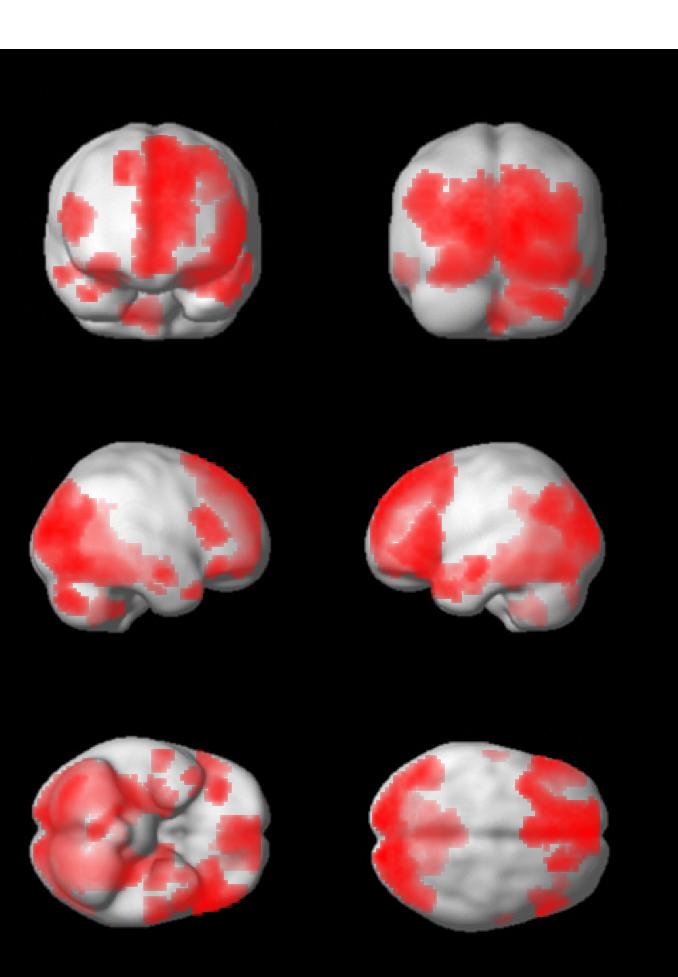


**Figure 2**

**Imagination task v control task: all participants (n=69)**


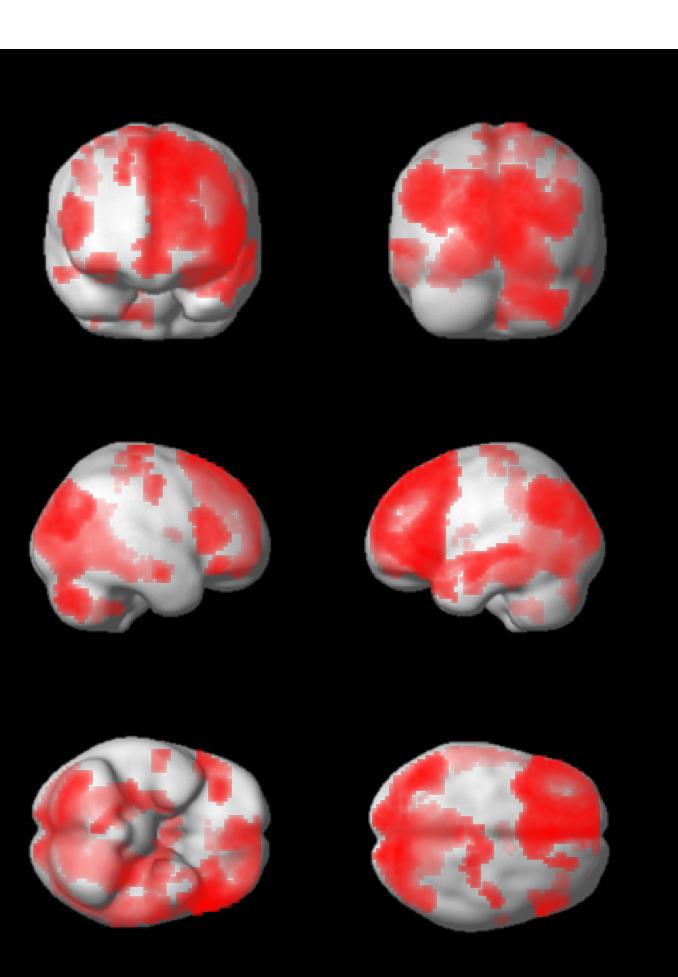


**Figure 3**

**Perception task v imagination task: all participants (n=69)**


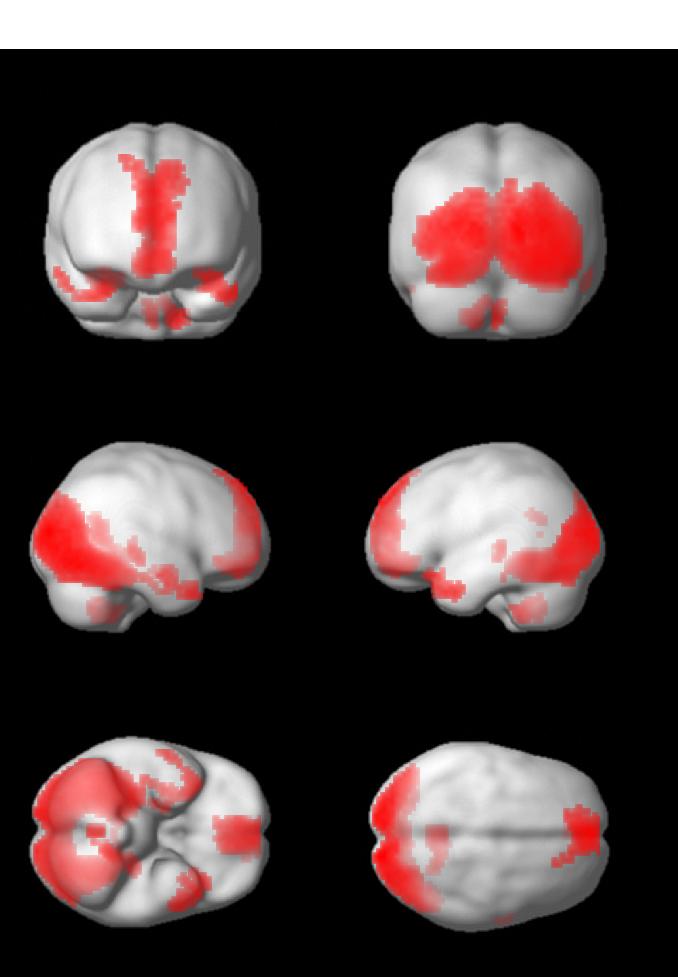


**Figure 4**

**Imagination task v perception task: all participants (n=69)**


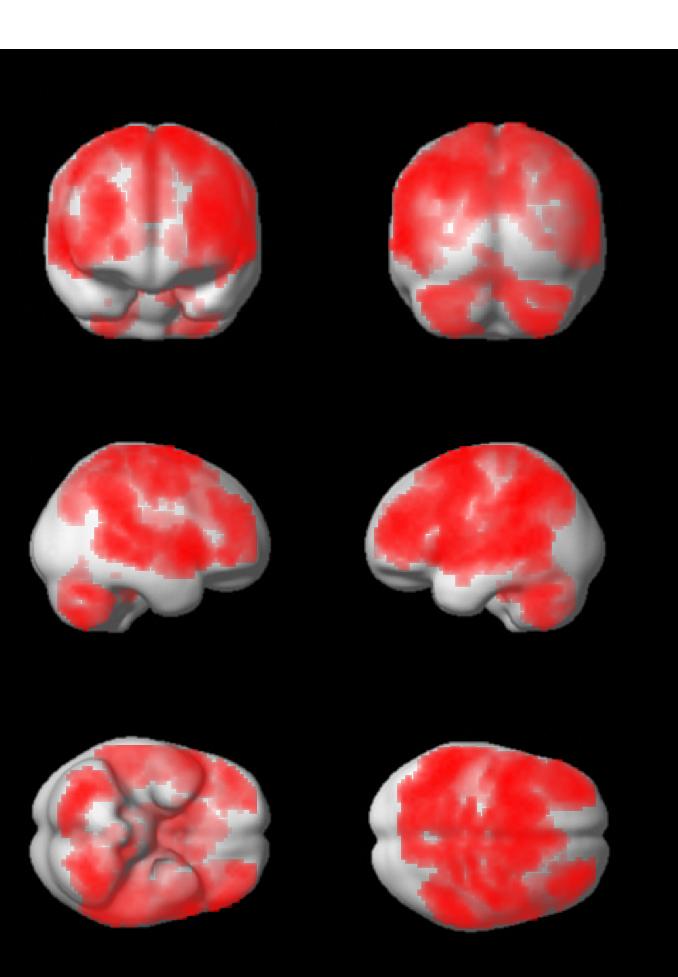


**Figure 5**

1. **Perception task v Control task: Individual Groups**


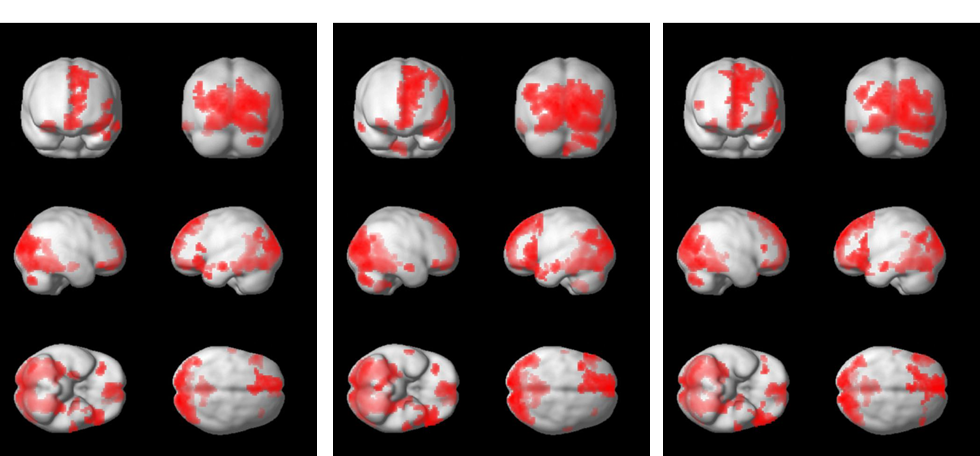


**Aphantasia (n=24) Hyperphantasia (n=25) Control (n=20)**

1. **Imagination task v Control task: Individual Groups**


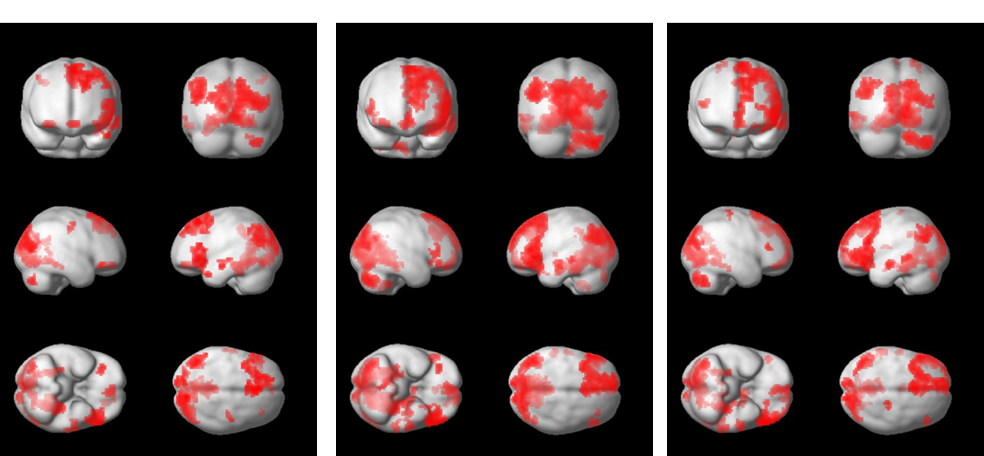


**Aphantasia (n=24) Hyperphantasia (n=25) Control (n=20)**
